# ﻿The morphology and spectral characteristics of the compound eye of *Agasicleshygrophila* (Selman & Vogt, 1971) (Coleoptera, Chrysomelidae, Galerucinae, Alticini)

**DOI:** 10.3897/zookeys.1177.100084

**Published:** 2023-08-30

**Authors:** Wei-Li Fan, Xiao-Kun Liu, Tian-Hao Zhang, Zu-Long Liang, Lei Jiang, Le Zong, Cong-Qiao Li, Zhong Du, Hao-Yu Liu, Yu-Xia Yang, Feng-Ming Wu, Si-Qin Ge

**Affiliations:** 1 Key Laboratory of Zoological Systematics and Evolution, Institute of Zoology, Chinese Academy of Sciences, Beichen West Road, Chaoyang District, Beijing 100101, China Institute of Zoology, Chinese Academy of Sciences Beijing China; 2 University of Chinese Academy of Sciences, Beijing 100049, China University of Chinese Academy of Sciences Beijing China; 3 The Key Laboratory of Zoological Systematics and Application, School of Life Science, Institute of Life Science and Green Development, Hebei University, Baoding 071002, Hebei Province, China Hebei University Baoding China

**Keywords:** Electroretinogram, insect vision, phototaxis, rhabdomere, 3D reconstruction

## Abstract

The first exploratory study was conducted on the compound eye morphology and spectral characteristics of *Agasicleshygrophila* (Selman & Vogt, 1971) to clarify its eye structure and its spectral sensitivity. Scanning electron microscopy, paraffin sectioning, and transmission electron microscopy revealed that *A.hygrophila* has apposition compound eyes with both eucones and open rhabdom. The micro-computed tomography (CT) results after 3D reconstruction demonstrated the precise position of the compound eyes in the insect’s head and suggested that the visual range was mainly concentrated in the front and on both sides of the head. The electroretinogram (ERG) experiment showed that red, yellow, green, blue, and ultraviolet light could stimulate the compound eyes of *A.hygrophila* to produce electrical signals. The behavioural experiment results showed that both males and females had the strongest phototaxis to yellow light and positive phototaxis to red, green, and blue light but negative phototaxis to UV light. This study of the compound eyes of *A.hygrophila* will be helpful for decoding its visual mechanism in future studies.

## ﻿Introduction

Compound eyes are the most prominent visual organ of most insects (Buschbeck and Friedrich 2008) and play a significant role in feeding, nesting, rhythm regulation, navigation, and other behaviours (Duelli and Wehner 1973; Helfrich-Förster et al. 2001; Cronin et al. 2003; Jia and Liang 2015; Ogueta et al. 2018). Compound eyes consist of ommatidia, whose number varies with insect species (Fischer et al. 2014). Ommatidia comprise five basic structures: the cornea, crystalline cone, rhabdom, basement membrane, and a number of pigment cells between the ommatidia. Generally, a positive correlation is reported between the ommatidial number, the radius curvature, and better vision. In addition, the arrangement and size of the ommatidia can also affect the visual field and resolution of the compound eye (Schwarz et al. 2011). The compound eyes consist of the apposition eye and the superposition eye, most diurnal insects, such as bees (Hymenoptera) and dragonflies (Odonata: Anisoptera), have apposition eyes that possess a high visual resolution and low photosensitivity (Land 1997), nocturnal insects, such as most moths (Lepidoptera), often have superposition eyes that are sensitive to light but have a low visual resolution (Fischer et al. 2014). The structure of compound eyes has an intricate relationship with insect behaviour and is also considered the best model for studying biological visual physiology and behavioural responses (Evangelin et al. 2015).

Compound eyes have a high regulating ability and can adapt to optical conditions ranging from 0 to 150000 lux (Meyer-Rochow and Nilsson 1999). Previous studies have shown that most insects can detect three primary light wavelengths and are mostly sensitive to blue (400–500 nm), long-wavelengths (480–600 nm), and UV light (300–400 nm). *Papilioxuthus* (Linnaeus, 1767) (Lepidoptera, Papilionidae) has six categories of photoreceptors (Briscoe and Chittka 2001; Koshitaka et al. 2008; Sharkey et al. 2017; van der Kooi et al. 2021; Xu et al. 2021), which can interact with different spectra to identify colours (Warrant and Nilsson 2006; Buschbeck and Friedrich 2008), inducing phototactic insect behaviour. For example, blue colour can induce the feeding behaviour of *Hycleusapicornis* (Guérin, 1847) (Coleoptera, Meloidae), yellow colour can induce the phototaxis of *Meligethesaeneus* (Fabricius, 1775) (Coleoptera, Nitidulidae), and red colour can cause *Megalagrionxanthomelas* (Selys-Longchamps, 1876) (Odonata, Coenagrionidae) to be aggressive (Lebesa et al. 2011; Doering et al. 2012; Schröder et al. 2018).

*Agasicleshygrophila* (Selman & Vogt, 1971) (Coleoptera: Chrysomelidae, Galerucinae) is a leaf beetle that feeds exclusively on *Alternantheraphiloxeroides* ((Mart.) Griseb, 1879); Caryophyllales, Amaranthaceae). We selected this beetle as our research model because this is diurnal insect and exhibits monophagous habit. *Alternantheraphiloxeroides* is an invasive species in China (Li 2016), and *Agasicleshygrophila* was introduced to control this weed. It has since become economically significant and maintains an ecological balance in China.

**Figure 1.**
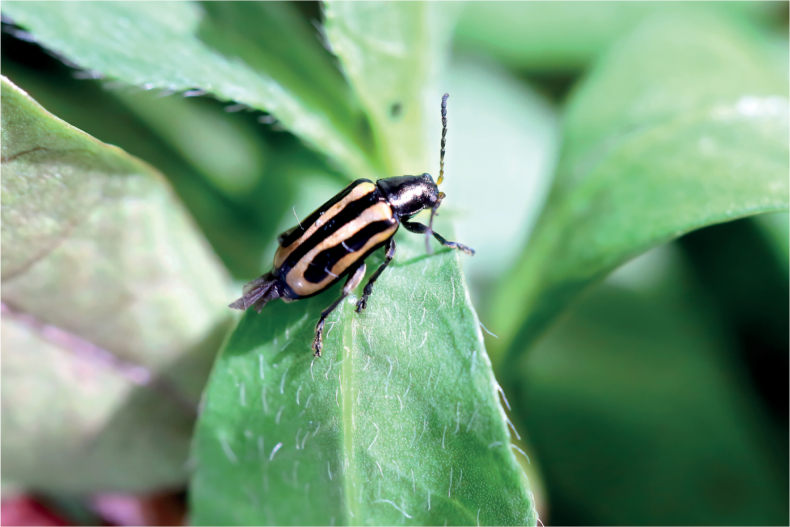
Adult male of *Agasicleshygrophila*.

Herbivorous insects locate host plants mainly by their vision and olfactory sensory organs. Li (2017) observed olfactory receptors using electron microscopy, but vision has not been widely studied. The aim of this study is to use vision and olfactory senses to find a more effective way to control *Agasicleshygrophila* (Fig. 1). We studied the visual system of this species in multiple dimensions by scanning electron microscopy, transmission electron microscopy, micro-computed tomography (CT), 3D reconstruction, and electroretinogram.

## ﻿Materials and methods

### ﻿Specimen collection

Live specimens of *A.hygrophila* were gifted from the Institute of Plant Protection, Chinese Academy of Agricultural Sciences and Shanxi Agricultural University. In the laboratory, adult male and female *A.hygrophila* were raised in an incubator with a controlled temperature of 25 ± 0.2 °C, relative humidity of 70 ± 5%, and photoperiod of 12:12 h. Leafy branches of the host plant, *Alternantheraphiloxeroides*, gifted from the Institute of Plant Protection, Chinese Academy of Agricultural Sciences, were provided. The females of *A.hygrophila* could lay eggs on the leaves, then these leaves with eggs were moved to a cleaned Petri dish underlaid with wet filter paper. Both larvae and adults of *A.hygrophila* were fed with *A.philoxeroides* leaves. Old leaves were replaced with fresh leaves, and the Petri dishes were cleaned daily to provide a more comfortable environment. Adult males and females were collected immediately after eclosion and moved to clean Petri dishes underlaid with wet filter paper, and the experiment was conducted three days later.

### ﻿Morphological terminology

The naming of compound eye structures and the method of continuous section follow Wen et al. (2022).

### ﻿Scanning electron microscopy (SEM)

To observe the compound eye external structure, nine male and female adult heads and prothoraxes were separated from the body and kept in water with 2.5% Tween 20 in a cryogenic vial, which was placed in a 40 kHz ultrasonic bath (KQ-50DE) for three cycles of 100 s of cleaning. After each cleaning, the sample was rinsed with distilled water for 20 s. After three cycles, a cleaned sample was obtained and then dehydrated with graded ethanol (once in 75%, 80%, 85%, 90%, and 95%, and 100% (3 × 30 min)). Then, the head and prethorax samples were dried using a critical-point dryer (Leica EM CPD 300), and later, with an electrically conductive adhesive were mounted on a rotatable specimen holder in a certain order and set with the desired spatial angle.

After sputter-coating with gold for 120 s (Leica EM SCD 050) from two different directions, the samples were examined with a scanning electron microscope (Leica FEI Quanta 450), and micrographs were captured at an accelerating speed of 5–15 kV.

### ﻿Light microscopy (LM)

The adult head was dehydrated in a series of ethanol (70%, 80%, 95%, and 100%) for 30 min at each concentration. After vitrification by xylene (2 × 20 min), the samples were embedded in paraffin twice for 30 min each. The series tissue sections (2 μm) were cut by microtome, dried in an oven, and then deparaffinised by xylene (2 × 10 min) each. Next, the stepwise staining of the sections was carried out as follows: 100% ethanol for 5 min, 95% ethanol for 5 min, 80% ethanol for 5 min, water for 5 min, haematoxylin staining solution for 5 min, water for 5 min, 1% ethanol hydrochloride for 3–5 s, water for 30 s, 1% ammonia for 10 s, water for 3 min, water for 1 min, 0.5% eosin staining solution (G1100-100, Beijing Solarbio Science & Technology Co., Ltd) for 1 min, water for 5 min, 95% ethanol (2 × 10 s), 100% ethanol (2 × 5 min), and xylene (2 × 5 min). After staining, the excess xylene was wiped off, environmentally friendly neutral gum was dropped on the tissue section, which was covered with a coverslip, and the order was marked. The images were observed and captured by an upright light microscope (Nikon Eclipse Ni-E) at a magnification of 20×.

### ﻿Transmission electron microscopy (TEM)

The adult head was fixed in fixation solution (2.5% (vol/vol) glutaraldehyde and 4% paraformaldehyde with phosphate buffer (PB)). Then, the tissues were fixed with 2.5% (vol/vol) glutaraldehyde and 1% tannic acid with phosphate buffer (0.1 M, pH 7.4), washed twice in PB and twice in double-distilled water (ddH_2_O). Then, the tissues were immersed in 1% (wt/vol) OsO_4_ and 1.5% (wt/vol) potassium ferricyanide aqueous solution at 4 °C for 2 h. After washing, tissues were dehydrated by washing with graded ethanol (30%, 50%, 70%, 80%, 90%, 100%, 100%, 10 min each) and pure acetone (2 × 10 min). Samples were infiltrated in graded mixtures (8:1, 5:1, 3:1, 1:1, 1:3, 1:5) of acetone and Spurr’s resin (10 g ERL 4221, 8 g DER 736, 25 g NSA, and 0.7% DMAE), and then pure resin. Finally, tissues were embedded in pure resin and polymerised for 12 h at 45 °C and 48 h at 70 °C. The ultrathin sections (70 nm thick) were sectioned with a microtome (Leica EM UC6), double-stained with uranyl acetate and lead citrate, and examined with a transmission electron microscope (FEI Tencai Spirit 120 kV). Micrographs were captured at an accelerating speed of 100 kV.

### ﻿Micro-computed tomography and 3D reconstruction

Decapitated samples were dehydrated in a series of graded ethanol 75%, 80%, 85%, 90%, 95%, and 3 ×100% (30 min in each concentration). After dehydration, the samples were dried in a freeze-dryer (Marin Christ) for 12 h, mounted on an Eppendorf tube and scanned using an X-radia scanner (Leica Micro XCT-400) at a magnification of 4×. A series image data set was captured at an interval of 9.0 s. 2D image stacks obtained through micro-CT scanning were reconstructed, and different compound eye structures were segmented by Amira software version 6.0.1. The segmented materials were imported into VG Studio Max 3.1 for rendering, polishing, colouring, and visualisation.

### ﻿Electroretinogram (ERG)

Three days after emergence, 12 male and female adults of *A.hygrophila* were selected for the ERG test. Appendages of samples were cut after 5 min of cryo-anaesthesia. A pair of glass electrodes fabricated using a micropipette puller were primed with conductive fluid (128.34 mM NaCl, 4.69 mM KCl, and 1.89 mM CaCl_2_·2H_2_O in water). A reference electrode was inserted into the abdomen intersegmental membrane, while the recording electrode was in contact with the compound eye surface. When the potential signal stabilised, five different light sources (red, yellow, green, blue, ultraviolet) were used for compound eye stimulation with three cycles of 10:10 s light:dark. After enlarging, the potential signal was recorded by a computer through WinWCP: Strathclyde Electrophysiology Software v. 5.1.1.1.

### ﻿Phototaxis test

For each light source set, three repeat tests were performed, with 20 samples for each test. The test chamber was improved from Kim et al.’s (2018) design (Fig. 9A), We designed a behaviour chamber with the different wavelength of light source on one side and *A.hygrophila* under free walking conditions in the middle of the box. Before light stimulation, samples were dark-adapted in the starting area for 20 min. Then, the light was turned on, and the visor was extracted for 5 min. Then, the number of individuals in the light area and dark area was counted. Ninety-five percent ethanol was used to clean the inner side of the chamber between each test. Red (620–625 nm), yellow (588–590 nm), green (515–525 nm), blue (460–465 nm), and ultraviolet (365–400 nm) light were used.

### ﻿Data analyses

ERG data were examined by Clampex software v. 10.6. The phototactic response was calculated by the following formula:

1. Positive phototaxis = (number of individuals in the light area/total individuals) × 100%
2. Negative phototaxis = (number of individuals in the dark area/total individuals) × 100%
3. Nonphototaxis = (number of individuals in the starting area/total individuals) × 100%

Analyses of the phototaxis data were performed with Origin 2021 and IBM SPSS Statistics 26 software. ANOVA and Tukey honestly significant difference (HSD) multiple comparisons were used to determine the significant differences between multiple groups. The significant difference between two treatments was determined using the independent-sample T test (Student’s t-test). Figures were produced with GraphPad Prism 8 software.

### ﻿Vouchers

The images of the SEM and microscope slides of serial sections are stored in the Institute of Zoology, Chinese Academy of Sciences.

## ﻿Results

### ﻿External morphology

**Figure 2.**
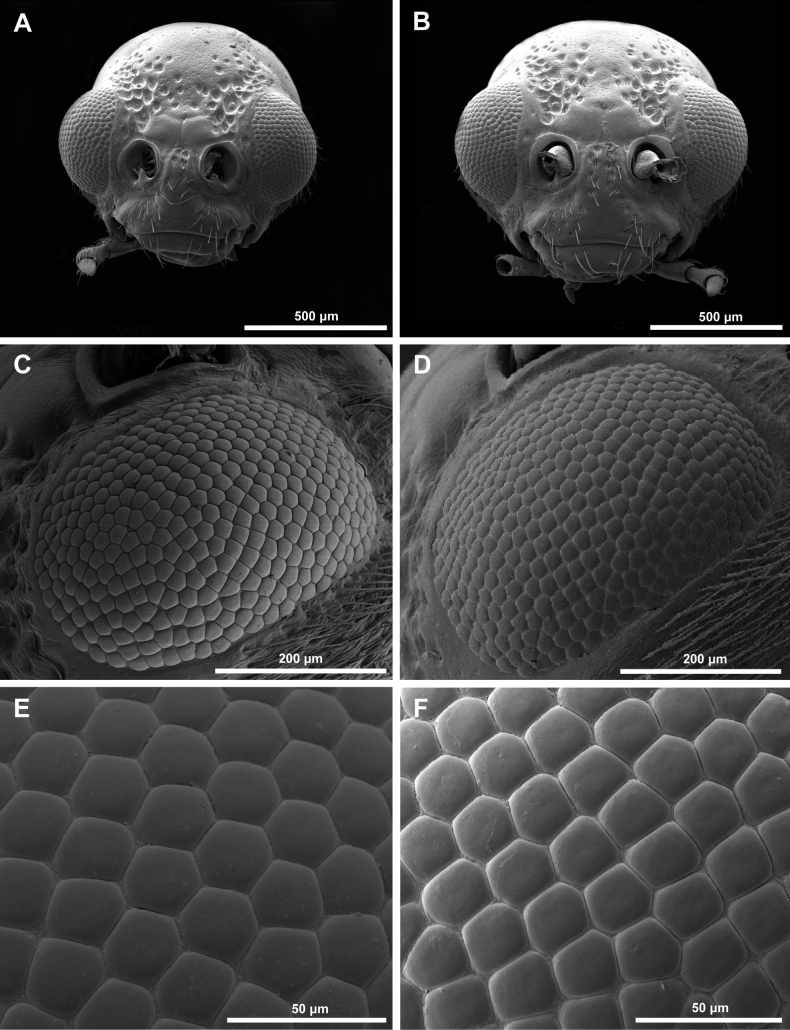
Scanning electron microscopy (SEM) of *Agasicleshygrophila***A** head of male **B** head of female **C** compound eye of male **D** compound eye of female **E** hexagonal facet **F** pentagonal facet. Scale bars: 50 μm (**E, F**); 200 μm (**C, D**); 500 μm (**A, B**).

Both male and female adults of *A.hygrophila* showed similar external morphological compound eye structures. The eyes are ellipsoid, located on both sides of the head, have a smooth surface, and exhibit no structural disparity (Figs 1, 2A, B). The number of ommatidia (341.00 ± 3.35 and 345.22 ± 4.48) was not significantly different between male and female compound eyes. The compound eyes lacked interommatidial hair between facets (Fig. 2C, D). Several facet shapes were observed: ommatidia in the central area of the compound eyes showed regular hexagonal facets, while ommatidia in the periphery of the compound eyes showed irregular shapes, mainly quadrilateral and pentagon (Fig. 2E, F).

### ﻿Internal morphology

**Figure 3.**
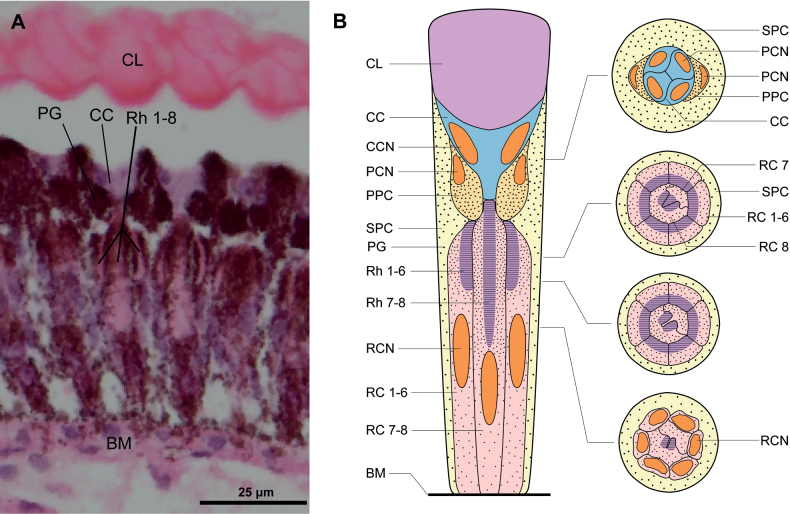
Diagram of one ommatidium of *Agasicleshygrophila***A** light micrographs (LM) of the compound eye of *Agasicleshygrophila***B** semi-schematic drawing of one ommatidium of *Agasicleshygrophila*. Abbreviations: CL: Corneal Lens; CC: Crystalline Cone; PG: Pigment Granule; Rh: Rhabdomere; BM: Basement Membrane; CCN: Cone Cell Nucleus; PPC: Primary Pigment Cell; PCN: Primary Pigment Cell Nucleus; SPC: Secondary Pigment Cell; RC: Retinular Cell; RCN: Retinular Cell Nucleus.

**Figure 4.**
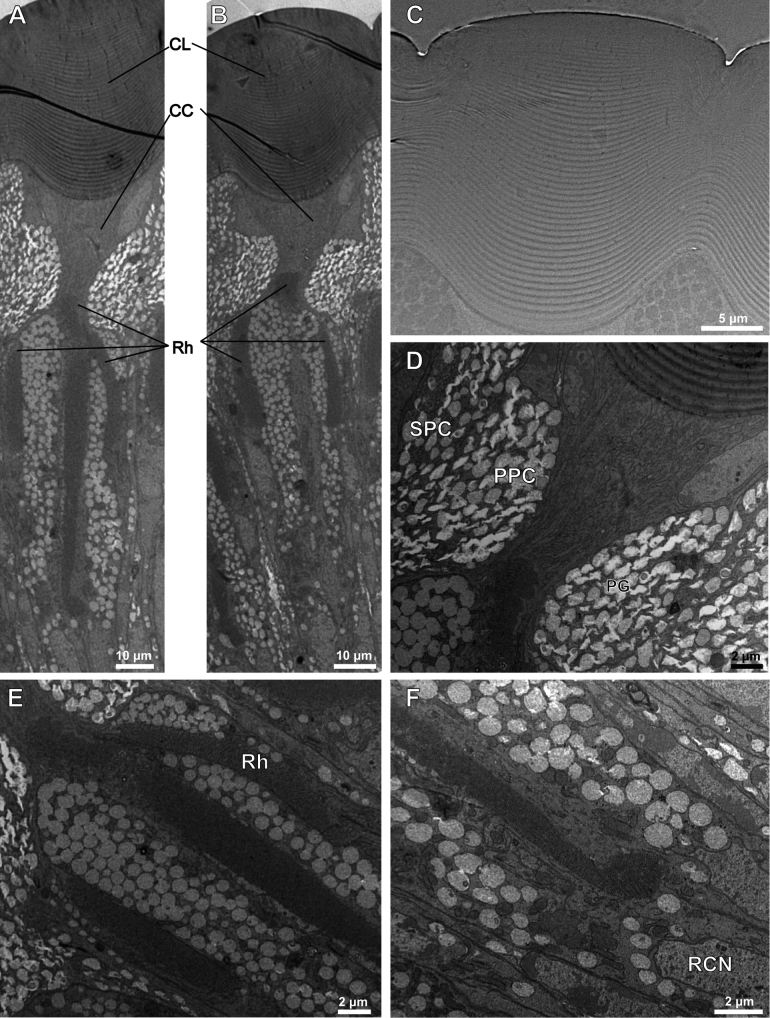
Transmission electron microscopy (TEM) of *Agasicleshygrophila***A, B** longitudinal section at different levels of the compound eye of *Agasicleshygrophila***C** corneal lens of one ommatidium of *Agasicleshygrophila***D** crystalline cone of one ommatidium of *Agasicleshygrophila***E, F** part of rhabdom of the compound eye of *Agasicleshygrophila*. Abbreviations: CL: Corneal Lens; CC: Crystalline Cone; Rh: Rhabdomere; BM: Basement Membrane; PPC: Primary Pigment Cell; SPC: Secondary Pigment Cell; RCN: Retinular Cell Nucleus.

We observed the internal morphological structure of adults of *A.hygrophila* compound eyes through a light microscope (Fig. 3) and transmission electron microscope. The ommatidium structures from the distal end to the proximal end are the corneal lens, crystalline cone, retinular cells with rhabdom, and basement membrane. The crystalline cone is surrounded by primary pigment cells, while secondary pigment cells spread around the entire ommatidium. We observed the corneal lens are directly in contact with the crystalline cone, with no clear zone. Therefore, we confirmed that *A.hygrophila* possesses apposition compound eyes (Fig. 4A, B).

**Figure 5.**
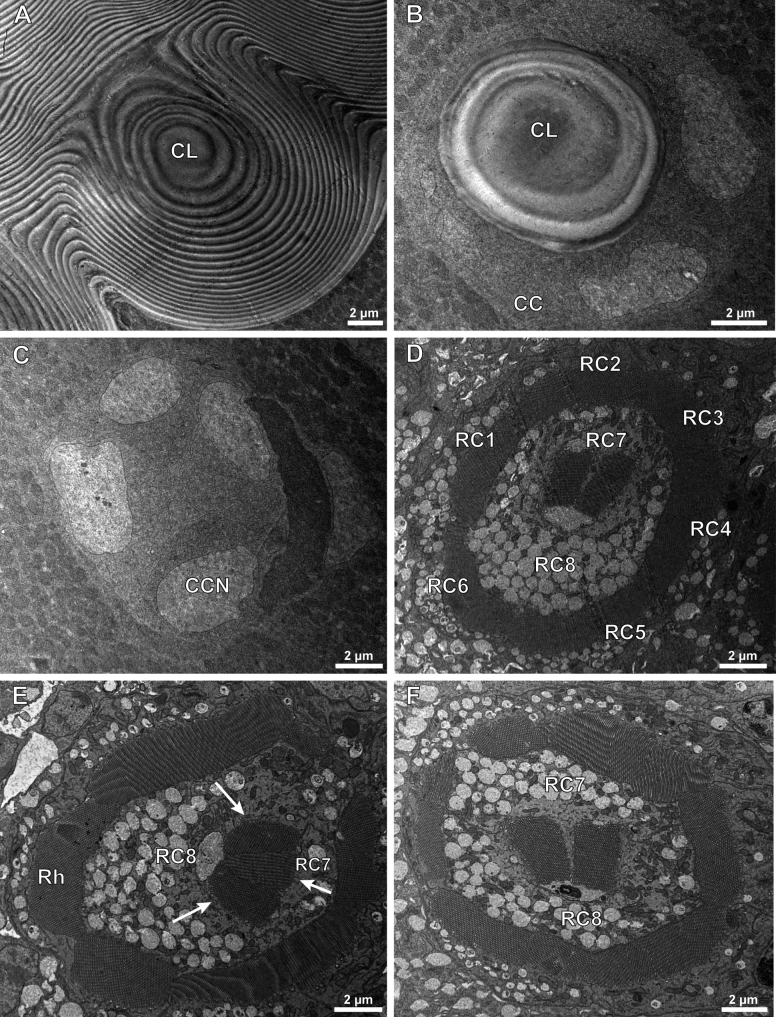
Transmission electron microscopy (TEM) of *Agasicleshygrophila***A** corneal lens of one ommatidium of *Agasicleshygrophila***B** corneal lens and crystalline cone of *Agasicleshygrophila***C** crystalline cone of *Agasicleshygrophila***D–F** transmission electron microscopy at different levels of the compound eye of *Agasicleshygrophila*. Abbreviations: CL: Corneal Lens; CC: Crystalline Cone; CCN: Cone Cell Nucleus; RC: Retinular Cell; Rh: Rhabdomere.

The corneal lens is the outermost structure of the ommatidium, with both outer and inner sides raised. It has a laminated structure composed of dense layers at the distal end and looser layers at the proximal end, ~ 60 layers with a total thickness of 25 μm (Fig. 4C). The cornea has a spiral shape in the cross-section, and the proximal end is surrounded by cone cells (Fig. 5A, B). Four wedge-shaped cone cells located just beneath the cornea are involved in forming the eucones. Each cone cell has a large, edge-located nucleus, which constitutes a quarter of the cone (Fig. 5C). The corneal lens and crystalline cone together form the dioptric apparatus.

Cell membranes near the longitudinal axis of the ommatidium are specialised to form rhabdomeres. Each ommatidium contains eight retinular cells, which means that each rhabdom is composed of eight rhabdomeres. Among them, six of all eight rhabdomeres form a wheel-shaped peripheral rhabdom, while the remaining two form a circular-shaped centred rhabdom. Thus, we confirm that *A.hygrophila* has an open rhabdom (Fig. 5D). We observed that two members of the centred rhabdom are not uniform in size but possess a strong rhabdom (Rh8) and a weak rhabdom (Rh7). The distal end containing Rh8 has a complete section, while the proximal end is divided into two parts by Rh7 (Fig. 5D, E). Rh7 is shorter than Rh8, and the distally centred rhabdom contains only Rh8 (Fig. 5F). However, the lengths of retinular cells 7 and 8 are the same.

### ﻿Three-dimensional reconstruction of the compound eye of *A.hygrophila*

**Figure 6.**
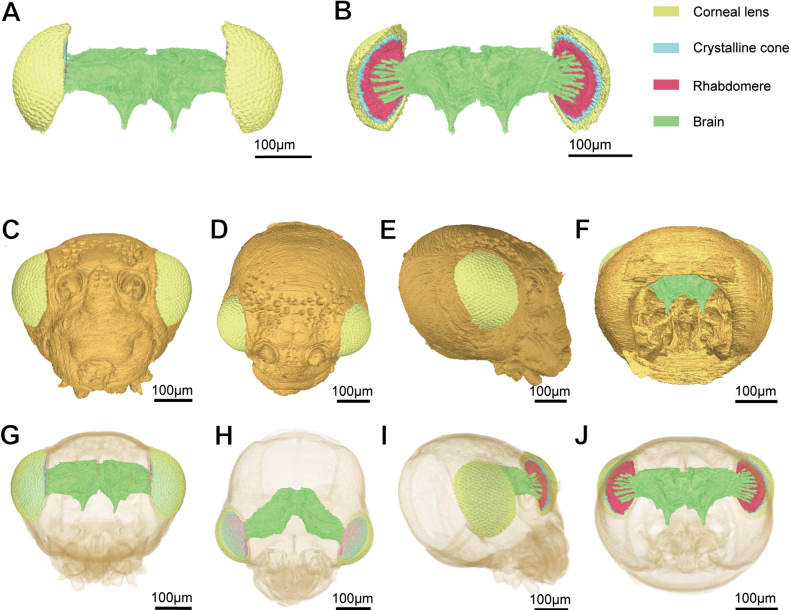
3D reconstruction of the head of *Agasicleshygrophila***A** front view of compound eye **B** rear view of compound eye **C** front view of head **D** top view of head **E** side view of head **F** rear view of head **G** perspective drawing of front view of head **H** perspective drawing of top view of head **I** perspective drawing of side view of head **J** perspective drawing of rear view of head.

In the reconstructed 3D image, the integral structure of the compound eyes and their location in the head are observed. Three distinguishable structures are shown (Fig. 6). The corneal lens in the outermost layer covered the crystalline cone, and the photoreceptive layer is beneath the cone layer. The retinular cells and pigment cells appeared to merge, the rhabdom tapers spanned from the distal to the proximal ends and finally connects with the brain (Fig. 6).

The distances from the compound eye to the front, top, bottom, and rear of the head were measured with the software Amira 6.0.1, with a distance ratio of 1:1.23:1.43:1.90. As a result, we determined that the compound eyes and vision range of *A.hygrophila* are located primarily on the front and sides of the head (Fig. 6C–J).

### ﻿Electroretinogram and phototaxis

**Figure 7.**
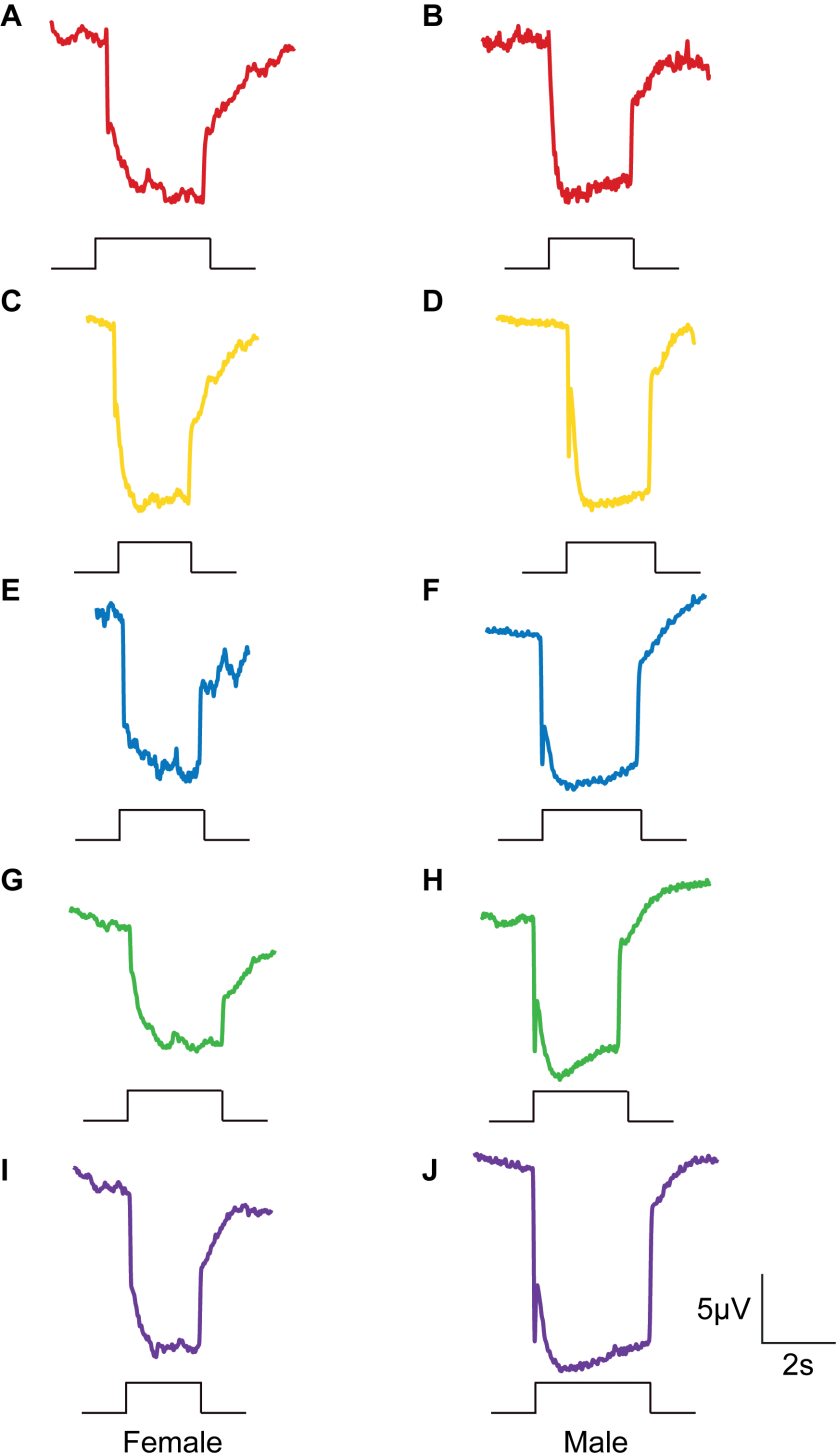
Electrophysiological waveforms of *Agasicleshygrophila* compound eyes at different wavelengths of light for females and males **A, B** red (620–630 nm) **C, D** yellow (588–590 nm) **E, F** blue (460–470 nm) **G, H** green (520–530 nm) **I, J** ultraviolet (365 nm).

**Figure 8.**
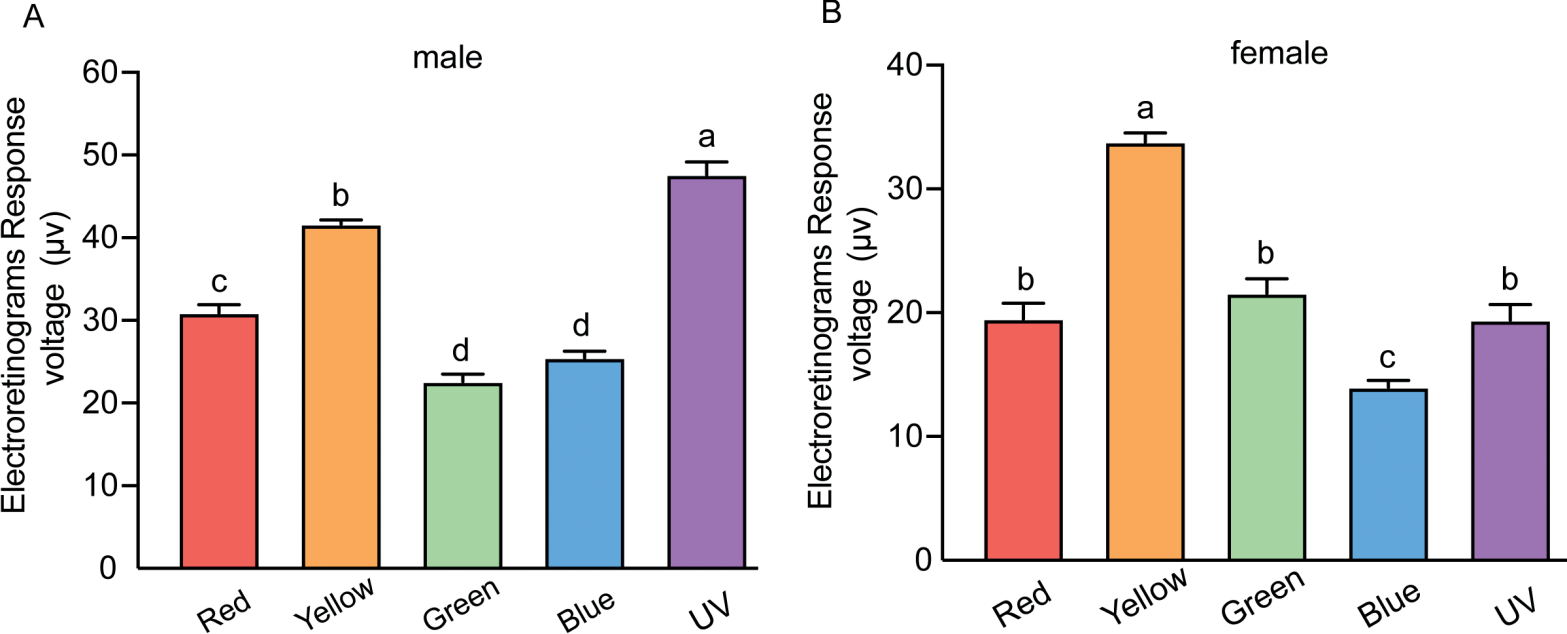
Quantification of Electroretinogram (ERG) voltage responses of *Agasicleshygrophila* that were exposed to a different light stimulus **A** responses of male **B** responses of female. Difference analysis was performed at the *P* < 0.05 level, and different letters indicate significant differences between ERG responses.

In the ERG experiments, the compound eyes of *A.hygrophila* emit signals after stimulation by five light colours (red, yellow, green, blue, and UV) (Fig. 7), but there are differences in the potentiometric responses between different light colours (Fig. 8). In this study, the signal intensity of the male *A.hygrophila* compound eyes generated by UV light stimulation is significantly higher than that generated by the other light colours, followed by that generated by yellow light. The responses of the male *A.hygrophila* compound eyes to blue light and green light are lower and not significantly different (Fig. 8A). In contrast, the signal intensity of female *A.hygrophila* compound eyes generated by yellow light is significantly higher than that generated by certain other light colours (red, green, blue, and UV), while that generated by the other light colours showed no significant difference. However, female *A.hygrophila* compound eyes are the least sensitive to blue light (Fig. 8B).

**Figure 9.**
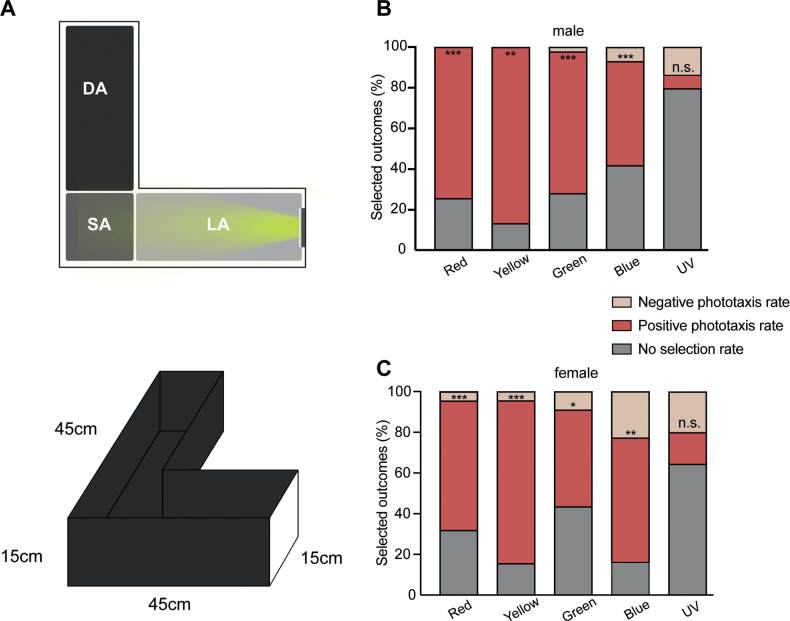
Results of behavioural experiments **A, B** behavioural experimental equipment **C** the phototaxis of male **D** the phototaxis of female. Data is presented as mean ± standard error of the mean (SEM). Abbreviations: DA: Dark Area, LA: Light Area, SA: Standing Area.

This study showed differences in the responses of the compound eyes of male and female *A.hygrophila* to five light colours (red, yellow, green, blue, and UV) via ERG experiments. However, we did not define here any differences in the phototaxis of males and females to the five lights. The male and female *A.hygrophila* showed significant phototaxis when exposed to red, yellow, green, and blue light, and the highest phototaxis was observed in yellow light, while avoidance was observed when UV light was used as the light source. Interestingly, the red colour also caused higher phototaxis in male and female *A.hygrophila* (Fig. 9).

## ﻿Discussion

In this study, SEM and TEM experiments revealed that that the compound eyes of *A.hygrophila* contains few ommatidia whose shape is irregular. There are without clear zone of compound eyes, this structure is a typical feature of apposition eyes. Through the ERG experiment, we found that there are a variety of photoreceptors in this species. The behaviour experiment showed that *A.hygrophila* has positive phototaxis to red, yellow, green, and blue light and has negative phototaxis to UV light.

The arrangement of microvilli affects the light perception of compound eyes. When the microvilli in the cross-section of the rhabdomere are arranged perpendicular to each other, there is a good possibility that the ommatidium could perceive polarised light. The rhabdomere abnormality leads to a decrease in light sensitivity (Labhart 1999). Our results from *A.hygrophila* compound eyes show that it has typical apposition compound eyes without clear zone and the microvillus of the rhabdomere are arranged irregularly. Therefore, we speculate that compound eyes of this species may be less sensitive to light.

There are significant differences in the surface structure and density of the ommatidium of different insects. Adult *A.hygrophila* feed and mate both during the day and night, and have a slightly convex ommatidium surface, which may increase the light contact area of the individual ommatidium and improve compound eye sensitivity. Numerous species of Coleoptera have six peripheral rhabdomeres and two central rhabdomeres, an arrangement pattern referred to as open rhabdom (Wachmann, 1979). The central rhabdomere and the peripheral rhabdomere are obviously separated from each other in *A.hygrophila*, resulting in an open rhabdom. The two central rhabdomeres in this ommatidium are arranged in a semi-enveloped structure, and the microvilli of the two rhabdomeres are not aligned in the same direction. Understanding the specific role of this structure in compound eye vision requires further investigation.

Most insects have at least two visual pigments, one detecting yellow–green light at ~ 550 nm wavelengths and the other detecting blue–violet light at wavelengths less than 480 nm (Briscoe and Chittka 2001). Previous studies found that the compound eyes of *Anomalacorpulenta* (Motschulsky, 1854) (Coleoptera, Scarabaeidae) are sensitive to near-UV, green–yellow, and blue light (Jiang et al. 2015). In our study, the ERG experiments showed that the compound eyes of *A.hygrophila* are sensitive to five light colours, red, yellow, green, blue, and UV, and they may have multiple visual pigments. Previous studies have found that beetles, such as *Thermonectusmarmoratus* (Gray, 1831) (Coleoptera, Dytiscidae) and *Triboliumcastaneum* (Herbst, 1797) (Coleoptera, Tenebrionidae), lack blue opsins, but there is evidence that the effects caused by the loss of blue opsins can be compensated by alternative mechanisms that restore sensitivity to blue light (Sharkey et al. 2017), such as in *Agrilusplanipennis* (Fairmaire, 1888) (Coleoptera, Buprestidae), which achieves sensitivity to blue light through the replication of other opsin genes. The compound eyes of *A.hygrophila* are sensitive to blue light, but this sensitivity may comprise abilities resulting from other opsin genes. Therefore, further molecular experiments are needed to verify this hypothesis.

Phytophagous insects, such as *M.aeneus*, *Diaphorinacitri* (Kuwayama, 1907) (Hemiptera, Liviidae), *Bactroceradorsalis* (Hendel, 1912) (Diptera, Tephritidae), and *Liriomyzahuidobrensis* (Blanchard, 1926) (Diptera, Agromyzidae), use yellow colour as a cue to find host plants (Bernays and Chapman 2007; Doering et al. 2012; Sétamou et al. 2014; Said et al. 2017). *Phyllotretastriolata* (Fabricius, 1801) (Coleoptera, Chrysomelidae) has strong phototaxis to UV and blue light (Yang et al. 2003); *Leptinotarsadecemlineata* (Say, 1824) (Coleoptera, Chrysomelidae) shows a strong response to green and yellow light (Otálora‐Luna and Dickens 2011). We found that both male and female *A.hygrophila* show strong phototaxis to yellow light, and yellow light may be an important cue for *A.hygrophila* to find host plants. In addition, blue light can stimulate phototaxis in some insects, such as *Frankliniellabispinosa* (Morgan, 1913) (Thysanoptera, Thripidae) (Childers and Brecht 1996). *Agasicleshygrophila* also showed significant phototaxis to blue light.

The wavelength of green colour is one of the common perceptive areas for most insects (van der Kooi et al. 2021). While insects can perceive green light, phototaxis to green light is low (Lebesa et al. 2011). Similarly, in this study, the phototaxis of *A.hygrophila* to green light is also relatively low among multiple green light colours, which may be related to the low green reflectance (43%) (Lebesa et al. 2011). Adult *A.hygrophila* prefer humidity and light avoidance (Wu et al. 2000), while sunlight contains a large amount of UV light, which may account for its low phototaxis to UV light (Fig. 9). Most beetles are insensitive to red light (van der Kooi et al. 2021); however, in this study we found that red light not only arouses the signal response of *A.hygrophila* compound eyes but also led to its phototaxis to red light. This characteristic may be related to the living environment of *A.hygrophila*: the adults mostly stay on leaves (Chen et al. 2009), and sunlight can appear as red light when shining through small gaps, while sunlight scattered in the sky at dusk also has a red spectrum (Endler 1993). Our hypothesis about the mechanisms of the perception of red light by *A.hygrophila* deserves further exploration.

## ﻿Additional information

### Author ORCIDs

Hao-Yu Liu https://orcid.org/0000-0003-1383-5560

Yu-Xia Yang https://orcid.org/0000-0002-3118-6659

### Data availability

All of the data that support the findings of this study are available in the main text or Supplementary Information.
